# Effects of athletic training on physical fitness and stroke velocity in healthy youth and adult tennis players: A systematic review and meta-analysis

**DOI:** 10.3389/fspor.2022.1061087

**Published:** 2023-01-10

**Authors:** Johanna Lambrich, Thomas Muehlbauer

**Affiliations:** Division of Movement and Training Sciences/Biomechanics of Sport, University of Duisburg-Essen, Essen, Germany

**Keywords:** intervention, muscle power/strength, agility, speed, balance, flexibility, endurance, stroke speed

## Abstract

Better physical fitness and stroke velocity in healthy elite compared to sub-elite tennis players have been shown in previous studies. However, evidence-based knowledge regarding the effectiveness of athletic training on physical fitness and stroke velocity is currently lacking. Thus, the objective of this systematic review with meta-analysis was to characterize, aggregate, and quantify athletic training effects on measures of physical fitness and stroke velocity in healthy youth and adult tennis players. A computerized systematic literature search was performed in the databases PubMed, Web of Science, and SportDiscus from their inception date to August 2022. Studies were included, among others, if the intervention period lasted a minimum of four weeks and if at least one parameter of physical fitness (i.e., speed, agility, lower-extremity muscle power, upper-extremity muscle power/strength, endurance, balance, flexibility) or stroke performance (i.e., stroke velocity) was tested. Initially, 11,511 articles were identified, after removing duplicates and assessing abstracts and full texts, 24 articles were used to calculate weighted standardized mean differences (*SMD*). For measures of physical fitness, athletic training resulted in small (speed: *SMD *= 0.44), moderate (endurance: *SMD *= 0.61, upper-extremity muscle power: *SMD *= 0.72; flexibility: *SMD *= 0.63), and large (agility: *SMD *= 0.93, lower-extremity muscle power: *SMD *= 0.88; upper-extremity muscle strength: *SMD *= 0.90; balance: *SMD *= 0.88) effects. Further, a large effect (*SMD *= 0.90) on stroke velocity was detected. The additionally performed sub-analyses showed differences in the effectiveness of athletic training on variables of physical fitness and stroke speed when considering players' age (i.e., youth players: <18 years; adult players: ≥18 years). Precisely, there was a high potential for training-related adaptations in adult players with respect to lower-extremity muscle power, upper-extremity muscle strength, and stroke velocity and in youth players with respect to endurance. Interventions to promote physical fitness and stroke velocity in healthy tennis players revealed varying levels of effectiveness ranging from small to large and these were additionally affected by players' age. Therefore, future studies should investigate modalities to increase training efficacy in youth and adult tennis players, especially for fitness components that showed small- to moderate-sized changes.

## 1. Introduction

Tennis is a popular sport and is characterized by high demands on both physical fitness (e.g., strength, power, agility, speed etc.) and technical (i.e., serve/stroke technique) factors. Both factors are used to distinguish successful from less successful players, which makes them particularly relevant for training purposes. In this regard, several cross-sectional studies (1–8) and a systematic review with meta-analysis (9) showed significant differences for both stroke velocity and the underlying physical fitness components (e.g., agility, muscle power, endurance, speed) in tennis players depending on their competition level. For example, Kramer et al. (1) detected shorter 10 m sprint times, better agility scores in the Spider test, and higher jump values in elite compared to sub-elite youth male and female players. Further, Ulbricht et al. (2) determined higher stroke velocities for the tennis serve in elite vs. sub-elite youth male and female players. Therefore, the question arises about the effectiveness of athletic training to increase variables of physical fitness and stroke performance in healthy tennis players. With regard to physical fitness, intervention studies with adult players showed beneficial effects on speed, muscle strength, and endurance (10, 11). However, a recent systematic review by Xiao et al. (12) reported a differentiated picture describing (a) significant performance improvements for speed and agility, (b) conflicting evidence regarding muscle power, and (c) no evidence with respect to muscle strength, flexibility, and endurance as a result of athletic training. These discrepancies between findings are most likely due to the fact that Xiao and colleagues only included studies with 12- to 18-year-olds, in whom processes of growth, maturation, and development are still ongoing compared to adults (13). Regarding stroke velocity, positive effects of athletic training were also reported in male youth and adult players (14, 15). Consistent with these findings from original studies, a recent review also reported training-related improvements in tennis serve velocity for the majority of included studies (16).

Although the aforementioned studies have increased the knowledge about the effects of athletic training programs on variables of physical fitness and stroke velocity in healthy youth tennis players, a systematic characterization, aggregation and, most importantly, quantification of the reported intervention effects especially for adult players is still lacking. Therefore, the purpose of the present systematic review with meta-analysis was to characterize, aggregate, and quantify the effects of athletic training on measures of physical fitness and stroke velocity in healthy youth (<18 years) and adult (≥18 years) tennis players. We assumed that athletic training leads to improvements in variables of physical fitness and stroke velocity, but effectiveness will differ with respect to of players’ age (i.e., youth vs. adult tennis players).

## 2. Methods

### 2.1. Search strategy

A systematic literature search of the PubMed, Web of Science, and SportDiscus databases was performed to identify eligible articles. The following Boolean expression was used:

Tennis AND ((training OR practice OR exercise OR intervention OR program OR drill) AND (functional OR performance OR agility OR flexibility OR athletic OR strength OR power OR speed OR fitness OR physical OR stroke OR balance OR resistance)) NOT table.

**Figure 1:**
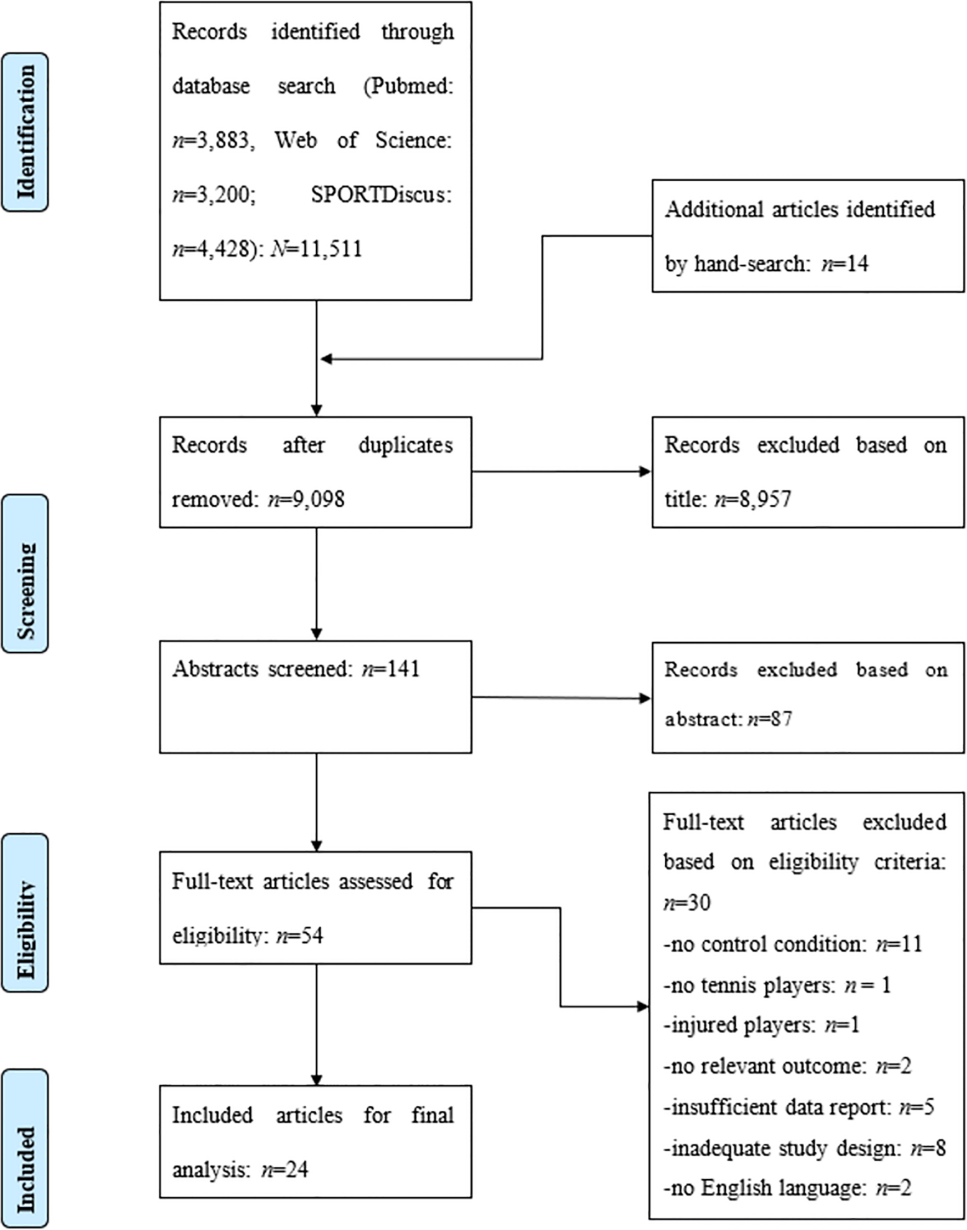
PRISMA flow chart illustrating the different phases of literature search, study selection, and reasons for exclusion of records.

The search covered the period between their inception date and August 2022. Only articles written in English with full-text access were included. In addition, the reference lists of included studies and relevant reviews were screened for relevant studies. After all duplicates were removed, both authors screened the title and abstract of all articles for eligibility according to the inclusion and exclusion criteria (Table 1). The full texts of all potentially eligible records were independently assessed by both authors and disagreements resolved through discussion and consents. The process of literature search, study selection, and exclusion criteria is presented in Figure 1 using the PRISMA flow chart (17).

**Table 1:** Overview of the inclusion and exclusion criteria.

Category	Inclusion criteria	Exclusion criteria
Population	Healthy female and male tennis players	Injured tennis players; no tennis players; beginner tennis players
Intervention	Athletic training; physical exercise; sport-specific intervention	Motor imagery; electromyostimulation
Comparison	Control group	No control condition
Outcome	At least one parameter of physical fitness or stroke velocity	Data did not allow to calculate effect size
Study design	Intervention studies with ≥4 weeks of training	Intervention studies with <4 weeks of training; no English full text

### 2.2. Study selection criteria

The inclusion and exclusion criteria are presented in Table 1. Studies were eligible for this review if they (a) examined healthy tennis players, (b) conducted an athletic or sport-specific intervention, (c) had a control condition, (d) reported at least one parameter of physical fitness or stroke velocity, and (e) performed an intervention that lasted at least four weeks as suggested by Farrell and Turgeon (18). Studies were excluded if (a) injured tennis players or no tennis players were studied, (b) the intervention consisted of non-physical exercises (e.g., motor imagery) or electromyostimulation was performed, (c) no control condition was present, (d) results did not allow the calculation of effect size, (e) acute effects (i.e., <4 weeks) of an intervention were studied, and (f) they were not published in English language.

### 2.3. Study coding

Included studies were coded using the following variables: author and year of publication, number of participants, sex, age, and study group with type of intervention. Interventions were coded based on the number of weeks of training, frequency and duration of a training session, and the number of sets and repetitions. If there were increases in volume during the intervention period, ranges were reported.

The following categories of physical fitness were differentiated: speed, agility, lower-extremity muscle power, upper-extremity muscle power/strength, endurance, balance, and flexibility. Further, stroke velocity was characterized *via* sport-specific assessments (i.e., serve test, forehand test). Because studies reported different parameters for each category, the most frequently reported measure was used to reduce heterogeneity between studies (Table 2).

**Table 2:** Overview of the preferred and alternative outcome by category.

Category	Preferred outcome	Alternative outcome
Speed	20 m sprint time in seconds (*n *= 5)	10 m sprint time seconds (*n *= 4)
Agility	5-0-5 agility test time in seconds (*n *= 4)	T-agility test in seconds (*n *= 2) Spider test in seconds (*n *= 2) Lateral agility test in seconds (*n *= 1) Illinois agility test in seconds (*n *= 1) Foran test in seconds (*n *= 1)
Lower-extremity muscle power	Countermovement jump height in cm (*n *= 6)	Vertical jump height in cm (*n *= 3)
Upper-extremity muscle power	Medicine ball throw in cm (*n *= 1)	seated medicine ball throw in cm (*n *= 1) Overhead medicine ball throw in km/h (*n *= 1)
Upper-extremity muscle strength	Handgrip strength (*n *= 3)	10-RM chest press in kg (*n *= 1) 1-RM bench press in kg (*n *= 1)
Endurance	VO_2_max in ml/min/kg (*n *= 2)	VIFT in km/h (*n* = 1) Wingate anaerobic power test in no. (*n* = 1) Ergometer test in Watt (*n *= 1)
Balance	Y-balance test in cm (*n *= 2)	N/A
Flexibility	Sit-and-reach test in cm (*n* = 2)	N/A
Stroke performance	Maximal stroke velocity in km/h (*n *= 14)	Mean stroke velocity in km/h (*n *= 1)

The figure in brackets indicates the number of studies that made use of the test. N/A, not available; RM, repetition maximum; VIFT, velocity of the intermittent fitness test.

### 2.4. Assessment of methodological quality

To assess the methodological quality of the included studies, the Physiotherapy Evidence Database (PEDro) scale was used (19). The PEDro scale rates study validity and statistical replicability of studies on a scale of 0 to 10, with ≥6 representing a cut-off score for high-quality studies (19). The predefined cut-off score of ≥6 points was not an inclusion or exclusion criterion. Quality assessment of the included studies was performed independently by both authors, and disagreements were resolved through discussion and consensus.

### 2.5. Statistical analyses

To quantify the effectiveness of athletic training programs on measures of physical fitness and stroke velocity, the within-subject standardized mean difference was calculated as *SMD*_W _= (pretest mean value – posttest mean value)/pretest standard deviation and the between-subject standardized mean difference as *SMD*_b _= (posttest mean value in the experimental group – posttest mean value in the control group)/pooled standard deviation (20) using Review Manager version 5.4.1. *SMD*_W_ and *SMD*_b_ can be positive or negative. Positive *SMD*_W_ values indicate an improvement in performance (i.e., increase in stroke velocity) from pretest to posttest, while negative *SMD*_W_ values indicate a decrease in performance (i.e., decrease in stroke velocity). Positive *SMD*_b_ values indicate an improvement in performance in favor of the experimental group (EG), while negative values indicate an improvement in favor of the control group (CG). The *SMD* values were reported for all players (6–42 years) as well as for youth (<18 years) and adult (≥18 years) players, separately.

*SMD*_W_ and *SMD*_b_ values can be classified and interpreted according to Cohen (21) into the following ranges: 0 ≤ 0.49 representing small effects, 0.50 ≤ 0.79 representing moderate effects, and ≥0.80 representing large effects. Further, heterogeneity (*I*^2^) was computed by using the formula provided by Deeks et al. (22): *I*^2^ = (*Q* – *df*/*Q*) * 100% where *Q* is the chi-squared statistics and *df* represents the degrees of freedom (23). This measure describes the percentage of the variability in effect estimates that is due to heterogeneity rather than sampling error (chance). Deeks et al. (22) postulate that heterogeneity can be interpreted as trivial (0 ≤ 40%), moderate (30 ≤ 60%), substantial (50 ≤ 90%), or considerable (75 ≤ 100%).

## 3. Results

### 3.1. Study selection

Figure 1 illustrates the different stages of the systematic literature search and the process of study selection. The search term resulted in 11,511 articles to be reviewed. In addition, 14 studies from other sources (i.e., reference lists, review articles) were added. After removing duplicates and screening titles and abstracts, 54 studies were screened for eligibility. Of these, 30 were excluded for the following reasons: eleven studies did not include a control group, one study did not examine tennis players, one study examined injured tennis players, two studies did not report relevant parameters (e.g., physical fitness, stroke velocity), five studies did not provide sufficient information on outcome measures, eight studies did not use an adequate study design, and two studies were not written in English language.

### 3.2. Study characteristics

Table 3 shows the characteristics of the 24 included studies. A total of 509 subjects aged between 6 and 42 years were investigated. Fifteen studies (14, 26, 28, 30, 31, 33–37, 39–43) examined youth tennis players under 18 years and nine reports (10, 11, 15, 24, 25, 27, 29, 32, 38) were conducted with adult tennis players aged between 18 and 42 years. Thirteen studies (14, 15, 24, 27–29, 31, 33, 34, 36, 39, 42, 43) investigated male tennis players, three studies (10, 11, 41) tested female players, five studies (25, 26, 30, 35, 40) examined both sexes while three studies (32, 34, 37) did not specify players' sex. Regarding performance level, four studies (10, 11, 25, 27) analyzed college players, three studies (28, 33, 34) examined national ranked players, two papers (24, 29) analyzed tournament players, one report each investigated competitive players (42), international ranked players (34), and ITN Level 3 (15). Twelve studies did not specify the players' performance level.

**Table 3:** Studies examining the effects of athletic training programs on measures of physical fitness and stroke velocity in healthy tennis players.

Reference	No. of subjects (sex); age [years (range or mean ± SD)]; performance level	Groups/training devices	Trainings modality No. of training weeks/frequency/sessions, single session duration, total duration per week No. of sets, reps, duration per exercise	Exercises	Test modality	Results
Mont et al. (24)	30, M; 18–42 years; tournament level	EG1 (*n *= 8): eccentric internal and external shoulder training EG2 (*n *= 9): concentric internal and external shoulder training CG (*n *= 13): no training	6 wk, 3 d, 18 sessions, N/A, N/A 8 sets of 10 reps	Isokinetic shoulder rotation	*Stroke performance:* Maximal serve velocity [km/h]	EG1-pp: $SMD_{W}=0.77$ EG2-pp: $SMD_{W}=1.86$ CG-pp: $SMD_{W}=0.13$ EG1-CG: $SMD_{b}=0.93\;(0.01,\;1.86)$ EG2-CG: $SMD_{b}=0.66\;(-0.22,\;1.53)$
Treiber et al. (25)	22, F (11), M (11); 21,2 years; college level	EG (*n *= 11): shoulder resistance training CG (*n *= 11): regular tennis training	4 wk, 3 d, 12 sessions, N/A, N/A 2–4 sets of 20 reps	TheraBand exercises (slow and quick); “Empty can” exercises with light dumbbell	*Stroke performance:* Maximal serve velocity [km/h]	EG-pp: $SMD_{W}=0.38$ CG-pp:$SMD_{W}=-0.12$ EG-CG: $SMD_{b}=0.25\;(-0.58,\;1.09)$
Kraemer et al. (10)	24, F; 17–21 years; college level	EG1 (*n *= 8): periodized training EG2 (*n *= 8): single-set circuit resistance training program CG (*n *= 8): regular tennis training	9 mo, 2–3 d, 100 sessions, 90 min; 180–270 min 14 exercises EG1: 2–4 sets of 4–6, 8–10 or 12-15 reps EG2: 1 set of 8–10 reps	Leg press, bench press, single curls, bent over rows, dumbbell lunge, split squat, military press, single knee, front pull downs, back extensions, internal/external rotations, sit-ups/crunches, hip tucks, wrist extensions/curls	*Lower-extremity muscle power:* Vertical jump height [cm]	EG1-pp: $SMD_{W}=1.02$ EG2-pp: $SMD_{W}=0.19$ CG-pp: $SMD_{W}=0.05$ EG1-CG: $SMD_{b}=0.54\;(0.14,\;2.27)$ EG2-CG: $SMD_{b}=0.07\;(-0.91,\;1.05)$
					*Upper-extremity muscle strength:* 1-RM bench press [kg]	EG1-pp: $SMD_{W}=1.42$ EG2-pp: $SMD_{W}=1.64$ CG-pp: $SMD_{W}=0.26$ EG1-CG: $SMD_{b}=2.13\;(0.90,\;3.36)$ EG2-CG: $SMD_{b}=1.78\;(0.62,\;2.94)$
					*Endurance:* Ergometer test [Watt]	EG1-pp: $SMD_{W}=0.80$ EG2-pp: $SMD_{W}=0.16$ CG-pp: $SMD_{W}=0.05$ EG1-CG: $SMD_{b}=1.37\;(0.28,\;2.46)$ EG2-CG: $SMD_{b}=0.26\;(-0.72,\;1.25)$
					*Stroke performance:* Maximal serve velocity [m/s]	EG1-pp: $SMD_{W}=-0.84$ EG2-pp: $SMD_{W}=-1.84$ CG-pp: $SMD_{W}=-0.41$ EG1-CG: $SMD_{b}=0.17\;(-0.79,\;1.12)$ EG2-CG: $SMD_{b}=0.06\;(-0.87,\;0.99)$
Kraemer et al. (11)	27, F; 19 ± 1 years; college level	EG1 (*n *= 9): non-linear periodized resistance training EG2 (*n *= 10): non-periodized resistance training CG (*n *= 8): regular tennis practice	9 mo, 2–3 d, 100 sessions, 90 min; 180–270 min 12 exercises EG1: 2–3 sets of 4–6, 8–10 or 12–15 reps EG2: 2–3 sets of 8–10 reps	Leg press, bench press, single curls, bent over rows, dumbbell lunge, split squat, military press, single knee extensions, internal/external rotations, sit-ups/crunches, hip tucks, wrist extensions/curls	*Endurance:* VO2max [ml/kg/min]	EG1-pp: $SMD_{W}=1.97$ EG2-pp: $SMD_{W}=0.14$ CG-pp: $SMD_{W}=-0.32$ EG1-CG: $SMD_{b}=2.51\;(1.20,\;3.82)$ EG2-CG: $SMD_{b}=0.68\;(-0.33,\;1.68)$
					*Speed:* 20 m sprint [s]	EG1-pp: $SMD_{W}=0.38$ EG2-pp: $SMD_{W}=0$ CG-pp: $SMD_{W}=0.41$ EG1-CG: $SMD_{b}=0.39\;(-0.58,\;1.35)$ EG2-CG: $SMD_{b}=0.29\;(-0.64,\;1.23)$
					*Agility:* Lateral agility test [s]	EG1-pp: $SMD_{W}=-0.38$ EG2-pp: $SMD_{W}=-0.14$ CG-pp: $SMD_{W}=0.16$ EG1-CG: $SMD_{b}=0.47\;(-0.50,\;1.43)$ EG2-CG: $SMD_{b}=0.25\;(-0.69,\;1.18)$
					*Upper-extremity muscle strength:* Handgrip strength [kg]	EG1-pp: $SMD_{W}\;0.64$ EG2-pp: $SMD_{W}\;0.74$ CG-pp: $SMD_{W}-0.34$ EG1-CG: $SMD_{b}\;1.12\;(0.09,\;2.14)$ EG2-CG: $SMD_{b}\;0.87\;(-0.10,\;1.84)$
					*Lower-extremity muscle power:* Vertical jump height [cm]	EG1-pp: $SMD_{W}=3.01$ EG2-pp: $SMD_{W}=2.17$ CG-pp: $SMD_{W}=0.51$ EG1-CG: $SMD_{b}=2.64\;(1.34,\;3.94)$ EG2-CG: $SMD_{b}=1.89\;(0.77,\;3.01)$
					*Stroke performance:* Maximal serve velocity [m/s]	EG1-pp: $SMD_{W}=1.95$ EG2-pp: $SMD_{W}=1.03$ CG-pp: $SMD_{W}=-0.41$ EG1-CG: $SMD_{b}=2.79\;(1.46,\;4.13)$ EG2-CG: $SMD_{b}=2.29\;(1.10,\;3.49)$
Malliou et al. (26)	40, both genders; 13–14 years; N/A	EG1 (*n *= 20): strength training CG (*n *= 20): regular tennis training	7 wk, 3 d, 21 sessions, 15 min; 45 min EG: 2–3 sets of 10–15 reps	EG1: 6 exercises	*Stroke performance:* Maximal serve velocity [km/h]	EG-pp: $SMD_{W}=0.53$ CG-pp: $SMD_{W}=0.09$ EG1-CG: $SMD_{b}=0.26\;(-0.36,\;0.88)$
Paul et al. (27)	20, M; 18–24 years; college level	EG1 (*n *= 10): agility training CG (*n *= 10): regular tennis training	EG: 7 wk, 4 d, 28 sessions, 30 min, 120 min 10 exercises	Ladder agility drill, lateral cone slalom, forward and backward cone slalom, spider run, cross cone, medicine ball mini-tennis	*Agility:* Illinois agility test [s]	EG1-pp: $SMD_{W}=0.79$ CG-pp: $SMD_{W}=0.12$ EG1-CG: $SMD_{b}=1.24\;(0.29,\;2.20)$
Fernandez-Fernandez et al. (28)	30, M; 14.2 ± 0.5 years; national ranked	EG (*n *= 15): strength training CG (*n *= 15): regular tennis training	6 wk, 3 d; 18 sessions, 60–70 min; 180–210 min 2 sets of 20 reps	EG: medicine ball exercises, core training, elbow extension, rowing, external rotation, shoulder abduction, diagonal pattern flexion, reverse throw, forward throw, wrist flexion/extension	*Stroke performance:* Maximal serve velocity [km/h]	EG-pp: $SMD_{W}=0.62$ CG-pp: $SMD_{W}=0.05$ EG-CG: $SMD_{b}=0.98\;(0.23,1.74)$
Behringer et al. (14)	36, M; 15.03 ± 1.64 years, N/A	EG1 (*n *= 12): resistance training EG2 (*n *= 12): plyometric training CG (*n *= 12): regular tennis training	8 wk, 2 d, 16 sessions, 90 min, 180 min EG1: 8 exercises, 2 sets of 15 reps EG2: 6–8 exercises, 3–4 sets of 10–15 reps	EG1: low pulley dead lifts, flexion abdominal machine, seated back-extension machine, lateral flexion machine, leg-press, chest-press, lat-pull-down machine EG2: rope skipping, lateral barrier hop, box hopping, CMJ, SJ, push-ups, medicine ball chest pass	*Stroke performance:* Mean serve velocity [km/h]	EG1-pp: $SMD_{W}=0.02$ EG2-pp: $SMD_{W}=0.30$ CG-pp: $SMD_{W}=-0.32$ EG1-CG: $SMD_{b}=0.04\;(-0.76,\;0.84)$ EG2-CG: $SMD_{b}=1.44\;(0.54,\;2.34)$
					*Upper-extremity muscle strength:* 10-RM chest press [kg]	EG1-pp: $SMD_{W}=0.80$ EG2-pp: $SMD_{W}=0.78$ CG-pp: $SMD_{W}=0.12$ EG1-CG: $SMD_{b}=0.56\;(-0.26,\;1.37)$ EG2-CG: $SMD_{b}=0.51\;(-0.30,\;1.33)$
Genevois et al. (15)	44, M; 26.9 ± 7.5 years; ITN 3	EG1 (*n *= 12): handled medicine ball training EG2 (*n *= 20): overweight racket training CG (*n *= 12): regular tennis training	6 wk, 2 d, 90 min, 180 min EG1: 6 sets of 6 exercises EG2: 7–10 sets	EG1: medicine ball exercises EG2: 10 crosscourt forehand drives with overweight racket	*Stroke performance:* Maximal forehand velocity [m/s]	EG1-pp: $SMD_{W}=0.57$ EG2-pp: $SMD_{W}=0.91$ CG-pp: $SMD_{W}=-0.48$ EG1-CG: $SMD_{b}=1.75\;(0.81,\;2.69)$ EG2-CG: $SMD_{b}=0.62\;(-0.20,\;1.44)$
Ölcücü et al. (29)	40, M, 20-25 years; tournament level	EG1 (*n *= 20): plyometric training CG (*n *= 20): regular tennis training	8wk, 3d, 35 min, 105 min 2 sets of 12 reps	N/A	*Stroke performance:* Maximal serve velocity [m/s]	EG-pp: $SMD_{W}=1.94$ CG-pp: $SMD_{W}=1.11$ EG-CG: $SMD_{b}=1.60\;(0.98,\;2.31)$
Sannicandro et al. (30)	23, F (8), M (15); 12–14 years, N/A	EG (*n *= 11): balance training CG (*n *= 12): tennis-specific drills	6 wk, 2 d, 12 sessions, 30 min, 60 min EG: 3–4 sets of 5–10 reps, 6 exercises	EG: high skipping, diagonal 1-legged bounds, maintaining equilibrium before the last bound for 3 s, forwards bounds equilibrium before the last bound for 3 s, low rows using an elastic exercises band bound to a support performed against bipodalic inflatable disk, medicine ball chest passes with balancing on a bipodalic inflatable disk or standing on one leg, exercises with unstable surface	*Speed:* 20 m sprint [s]	EG-pp: $SMD_{W}=0$ CG-pp: $SMD_{W}=0$ EG-CG: $SMD_{b}=0.39\;(-0.58,\;1.35)$
					*Agility:* Foran test [s]	EG-pp: $SMD_{W}=0.33$ CG-pp: $SMD_{W}=0$ EG-CG: $SMD_{b}=1.42\;(0.46,\;2.38)$
Fernandez-Fernandez et al. (31)	16, M, 16.9 ± 0.5 years; international ranked	EG (*n *= 8): combined explosive strength and repeated sprint training CG (*n *= 8): regular tennis training	8 wk, 3 d, 50–80 min, 150–240 min EG: sprint training: 3–4 sets of 5–6 reps explosive strength: 4–6 exercises with 3–4 sets of 12–15 reps	15-20 m sprint, CMJ, multilateral hops, plyometric jumps, step multilateral calf jumps, agility drills, resisted standing start sprints	*Speed:* 20 m sprint [s]	EG-pp: $SMD_{W}=0.50$ CG-pp: $SMD_{W}=-0.20$ EG-CG: $SMD_{b}=0.90\;(-0.13,\;1.93)$
					*Lower-extremity muscle power:* CMJ height [cm]	EG-pp: $SMD_{W}=0.43$ CG-pp: $SMD_{W}=-0.11$ EG-CG: $SMD_{b}=1.73\;(0.58,\;2.88)$
					*Endurance:* VIFT [km/h]	EG-pp: $SMD_{W}=0$ CG-pp: $SMD_{W}=0.33$ EG-CG: $SMD_{b}=0\;(-0.98,\;0.98)$
Kara et al. (32)	20, N/A; 18-24 years; N/A	EG (*n *= 10): tennis specific strength training CG (*n *= 10): regular tennis training	6 wk; 3 d; 18 sessions; 45–60 min; 135–180 min 11 exercises, 3–4 sets of 8–12 reps/30 s	Squat, single arm, rotational service pull, isometric quarter squat, tennis serves shot throw, 2 arm 90/90 external rotation, reverse 90/90 throw, plyometric 90/90 internal rotation in service position, jump into single-leg Romanian dead lift, crunch, throwing medicine ball, jump at squat position and static stance	*Stroke performance:* Maximal serve velocity [km/h]	EG-pp: $SMD_{W}=1.85$ CG-pp: $SMD_{W}=0.54$ EG-CG: $SMD_{b}=-0.56\;(-1.46,\;0.33)$
Fernandez-Fernandez et al (33)	60, M; 12.5 ± 0.3 years; national ranked	EG (*n *= 30): plyometric training for the upper and lower body CG (*n *= 30): regular tennis training	8 wk, 2 d, 16 sessions, 30–60 min, 60–120 min 4–8 exercises, 2–4 sets of 10–15 reps	Medicine ball throws, CMJ, 2/1-leg zigzag over lines, 2-leg multidirectional hurdle jumps, lateral bounds and stabilization, 1-foot ankle hop forward, 1-leg box jump	*Lower-extremity muscle power:* CMJ height [cm]	EG-pp: $SMD_{W}=0.44$ CG-pp: $SMD_{W}=0.14$ EG-CG: $SMD_{b}=0.27\;(-0.24,\;0.78)$
					*Speed:* 20 m sprint [s]	EG-pp: $SMD_{W}=0.65$ CG-pp: $SMD_{W}=0.05$ EG-CG: $SMD_{b}=0.76\;(0.23,\;1.28)$
					*Agility:* 505 agility test [s]	EG-pp: $SMD_{W}=0.45$ CG-pp: $SMD_{W}=0.10$ EG-CG: $SMD_{b}=-0.38\;(-0.13,\;0.89)$
					*Upper-extremity muscle power:* MBT [cm]	EG-pp: $SMD_{W}=0.59$ CG-pp: $SMD_{W}=0.03$ EG-CG: $SMD_{b}=0.70\;(0.17,\;1.22)$
					*Stroke performance:* Maximal serve velocity [km/h]	EG-pp: $SMD_{W}=1.06$ CG-pp: $SMD_{W}=0.10$ EG-CG: $SMD_{b}=0.56\;(0.04,\;1.07)$
Fernandez-Fernandez et al. (34)	20, N/A; 14.8 ± 0.1 years; national ranked	EG (*n *= 10): mixed high intensity intermittent runs and tennis specific training CG (*n *= 10): tennis-specific drills	8 wk; 2 d; 16 sessions; 16-22 min, 32–44 min 2 sets of 8–11 min runs	Big x, suicide, recovery/defense, open pattern	*Speed:* 20 m sprint	EG-pp: $SMD_{W}=-0.42$ CG-pp: $SMD_{W}=-0.32$ EG-CG: $SMD_{b}=-0.08\;(-0.96,\;0.80)$
					*Agility:* 505-agility test [s]	EG-pp: $SMD_{W}=1.00$ CG-pp: $SMD_{W}=0.12$ EG-CG: $SMD_{b}=-0.63\;(-0.27,\;1.53)$
					*Lower-extremity muscle power:* CMJ height [cm]	EG-pp: $SMD_{W}=0.25$ CG-pp: $SMD_{W}=0.50$ EG-CG: $SMD_{b}=0.10\;(-0.78,\;0.97)$
					*Endurance:* VO_2_max [ml/kg/min]	EG-pp: $SMD_{W}=1.13$ CG-pp: $SMD_{W}=0.55$ EG-CG: $SMD_{b}=0.87\;(-0.05,\;1.78)$
Terrazo-Rebollo et al. (35)	20, F (5), M (15); 15.5 ± 0.9 years, N/A	EG1 (*n *= 7): regular tennis training and training with overloads EG2 (*n *= 7): regular tennis training and medicine ball throws and elastic bands CG (*n *= 6): regular tennis training	8 wk, 3 d, 60 min, 180 min per session (120 min tennis and 60 min additional training) EG1: 9 exercises, 3 sets with 6-14 reps EG2: 8 exercises, 3 sets of 6 reps	EG1: bench press, trunk curl, leg press, forehand/backhand barbell, trunk extension, dumbbell lying shoulder external rotation, one arm dumbbell row to waist, standing high pulley internal rotation, barbell throw, squat EG2: forehand/backhand side throws, chest throws, two-arm overhand forward/backhand throws, one-arm overhead forward throws, side floor throws, two-arm trunk rotation, one-arm diagonal trunk flexion	*Stroke performance:* Maximal serve velocity [km/h]	EG1-pp: $SMD_{W}=0.42$ EG2-pp: $SMD_{W}=0.14$ CG-pp: $SMD_{W}=-0.11$ EG1-CG: $SMD_{b}=0.24\;(-0.86,\;1.33)$ EG2-CG: $SMD_{b}=0.18\;(-0.91,\;1.27)$
Yildiz et al. (36)	28, M; 9.6 ± 0.7 years; N/A	EG1 (*n *= 10): traditional training EG2 (*n *= 10): functional training CG (*n *= 8): regular tennis training	8 wk, 3 d; 24 sessions; 70 min; 210 min 10 exercises, 3 sets of 10–12 reps	EG1: chest press, shoulder press, lateral pull-down, biceps curls, triceps push-down, seated leg extensions, leg curl, standing calf rise, modified push-up, sit-up EG2: squat, dead bug, plank, bridge, chop, lift, push up, pull up, medicine ball throw	*Lower-extremity muscle power:* CMJ height [cm]	EG1-pp: $SMD_{W}=0.24$ EG2-pp: $SMD_{W}=1.81$ CG-pp: $SMD_{W}=0.23$ EG1-CG: $SMD_{b}=1.21\;(0.20,\;2.22)$ EG2-CG: $SMD_{b}=2.66\;(1.38,\;3.93)$
					*Speed:* 10 m sprint [s]	EG1-pp: $SMD_{W}=-0.18$ EG2-pp: $SMD_{W}=1.55$ CG-pp: $SMD_{W}=0.23$ EG1-CG: $SMD_{b}=1.75\;(0.81,\;2.69)$ EG2-CG: $SMD_{b}=0.62\;(-0.20,\;1.44)$
					*Agility:* T-agility test [s]	EG1-pp: $SMD_{W}=-0.08$ EG2-pp: $SMD_{W}=1.11$ CG-pp: $SMD_{W}=0$ EG1-CG: $SMD_{b}=0.64\;(-1.60,\;0.31)$ EG2-CG: $SMD_{b}=1.41\;(-2.45,\;-0.38)$
					*Balance:* Y-balance test [cm]	EG1-pp: $SMD_{W}=0.25$ EG2-pp: $SMD_{W}=3.06$ CG-pp: $SMD_{W}=-0.12$ EG1-CG: $SMD_{b}=0.53\;(-0.41,\;1.48)$ EG2-CG: $SMD_{b}=3.06\;(1.70,\;4.43)$
					*Flexibility:* Sit-and-reach test [cm]	EG1-pp: $SMD_{W}=0$ EG2-pp: $SMD_{W}=1.71$ CG-pp: $SMD_{W}=0$ EG1-CG: $SMD_{b}=0.57\;(-0.38,\;1.52)$ EG2-CG: $SMD_{b}=2.78\;(1.48,\;4.08)$
Bashir et al. (37)	30, N/A; 15.3 ± 0.8; N/A	EG (*n *= 15): core training CG (*n *= 15): control group	5 wk, 3 d, 15 sessions, N/A, N/A 3–5 exercises, 1–3 sets of 15–20 reps	Supine and quadruped abdominal muscle contraction, side bridge, dead bug supine, medicine ball rotation, squat, superman, oblique pulley with side shuffles, standing wall cross toss, diagonal curl, twist on a Swiss ball, single leg standing on unstable surface	*Agility:* T-agility test [s]	EG-pp: $SMD_{W}=0.59$ CG-pp: $SMD_{W}=-0.19$ EG-CG: $SMD_{b}=1.31\;(0.52,\;2.10)$
Ziagkas et al. (38)	24, M, 20.9 ± 0.7years	EG (*n *= 12): plyometric training CG (*n *= 12): watching tennis matches	8 wk, 2d, 16 sessions, 30 min, 60 min N/A, 2-4 sets of 10-15 reps	N/A	*Agility:* Spider-Test [s]	EG-pp: $SMD_{W}=2.06$ CG-pp: $SMD_{W}=0.64$ EG-CG: $SMD_{b}=2.27\;(0.63,2.45)$
Kocyigit et al. (39)	24, M; 12–14 years, N/A	EG (*n *= 12): combined training CG (*n *= 12): regular tennis training	3 mo, 3 d, 36 sessions, 90 min, 270 min	EG: strength, speed, agility, endurance training; rope skipping, rally exercises	*Stroke performance:* Maximal serve velocity [km/h]	EG-pp: $SMD_{W}=2.06$ CG-pp: $SMD_{W}=0.77$ EG-CG: $SMD_{b}=2.89\;(1.74,\;4.03)$
Egesoy et al. (40)	36, F (15), M (21); 10–14 years, N/A	EG1 (*n *= 12): static core training EG2 (*n *= 12): dynamic core training CG (*n *= 12): regular tennis training	8 wk, 2d, 16 sessions, 25–30 min, 50–60 min 9 exercises, 2 sets of 20–40 s	EG1: front plank, side plank, leg raise hold, dead bug, bird and dogs, banana, superman, posterior plank, glute bridge hold EG2: plank climbers, supine plank leg lift, Russian twist, dead bug. Bird and dogs, leg raise, side plank, leg lift, plank leg extensions, reverse crunch	*Lower-extremity muscle power:* CMJ height [cm]	EG1-pp: $SMD_{W}=0.33$ EG2-pp: $SMD_{W}=0.58$ CG-pp: $SMD_{W}=0.09$ EG1-CG: $SMD_{b}=0.11\;(-0.70,\;0.91)$ EG2-CG: $SMD_{b}=-0.17\;(-0.98,\;0.63)$
					*Flexibility:* Sit-and-reach test [cm]	EG1-pp: $SMD_{W}=0.09$ EG2-pp: $SMD_{W}=0.06$ CG-pp: $SMD_{W}=0.24$ EG1-CG: $SMD_{b}=-0.14\;(-0.94,\;0.66)$ EG2-CG: $SMD_{b}=-0.42\;(-1.23,\;0.39)$
					*Upper-extremity muscle strength:* Handgrip strength [kg]	EG1-pp: $SMD_{W}=0.71$ EG2-pp: $SMD_{W}=0.78$ CG-pp: $SMD_{W}=0.10$ EG1-CG: $SMD_{b}=0.48\;(-0.33,\;1.29)$ EG2-CG: $SMD_{b}=0.30\;(-0.51,\;1.10)$
					*Speed:* 10 m sprint [s]	EG1-pp: $SMD_{W}=0.08$ EG2-pp: $SMD_{W}=0.06$ CG-pp: $SMD_{W}=0$ EG1-CG: $SMD_{b}=0.47\;(-0.34,\;1.28)$ EG2-CG: $SMD_{b}=0.29\;(-0.52,1.09)$
					*Balance:* Y-balance test [cm]	EG1-pp: $SMD_{W}=0.31$ EG2-pp: $SMD_{W}=0.37$ CG-pp: $SMD_{W}=0.12$ EG1-CG: $SMD_{b}=0.38\;(-0.43,\;1.19)$ EG2-CG: $SMD_{b}=0.04\;(-0.76,\;0.84)$
					*Upper-extremity muscle power:* Seated medicine ball throw test [cm]	EG1-pp: $SMD_{W}=0.20$ EG2-pp: $SMD_{W}=0.31$ CG-pp: $SMD_{W}=0.07$ EG1-CG: $SMD_{b}=0.46\;(-0.35,\;1.27)$ EG2-CG: $SMD_{b}=0.37\;(-0.44,\;1.18)$
					*Stroke performance:* Maximal serve velocity [km/h]	EG1-pp: $SMD_{W}=0.19$ EG2-pp: $SMD_{W}=0.19$ CG-pp: $SMD_{W}=0.03$ EG1-CG: $SMD_{b}=0.24\;(-0.56,\;1.05)$ EG2-CG: $SMD_{b}=0.16\;(-0.64,\;0.96)$
Zirhli et al. (41)	20, F; 10–12 years; N/A	EG (*n *= 10): 2 d tennis training, 2 d functional training CG (*n *= 10): 4 d tennis training	8 wk, 4 d, 90 min, 360 min EG: 9 exercises, 3 sets of 7–10 reps	EG: burpees, bungee run, forward jump squat, plank, torso rotation, side to side run, agility run, side to side crash, reverse walking	*Speed:* 10 m sprint [s]	EG-pp: $SMD_{W}=0.71$ CG-pp: $SMD_{W}=-0.13$ EG-CG: $SMD_{b}=1.81\;(0.77,\;2.85)$
					*Lower-extremity muscle power:* Vertical jump height [cm]	EG-pp: $SMD_{W}=0.52$ CG-pp: $SMD_{W}=0.03$ EG-CG: $SMD_{b}=1.01\;(0.08,\;1.94)$
					*Agility:* T-agility test [s]	EG-pp: $SMD_{W}=2.52$ CG-pp: $SMD_{W}=0.07$ EG-CG: $SMD_{b}=1.81\;(0.77,\;2.85)$
					*Upper-extremity muscle strength:* Handgrip strength [kg]	EG-pp: $SMD_{W}=0.96$ CG-pp: $SMD_{W}=0.02$ EG-CG: $SMD_{b}=1.43\;(0.45,\;2.41)$
					*Endurance:* Wingate anaerobic power test [no.]	EG-pp: $SMD_{W}=2.01$ CG-pp: $SMD_{W}=-0.11$ EG-CG: $SMD_{b}=1.74\;(0.71,\;2.77)$
					*Flexibility:* Sit-and-reach test [cm]	EG-pp: $SMD_{W}=0.57$ CG-pp: $SMD_{W}=0.13$ EG-CG: $SMD_{b}=0.83\;(-0.09,\;1.74)$
Canos et al. (42)	24, M; 14–16 years; competitive	EG1 (*n *= 8): machine-based training EG2 (*n *= 8): flywheel training CG (*n *= 8): regular tennis training	8 wk, 2 d, 16 sessions, N/A, N/A EG1 and EG2:5–6 exercises 3 sets of 6–8 reps followed by a block of 2–3 sets of 5–6 specific exercises	EG1: shoulder press, lateral pulldown, complete leg press, bench press, half squat, forward lunge, dumbbell power EG2: low row 90°, forehand closed stance, backhand stance, one handed chest crossover, one handed low row with one step, global chest press, one handed shoulder press	*Lower-extremity muscle power:* CMJ height [cm]	EG1-pp: $SMD_{W}=0.79$ EG2-pp: $SMD_{W}=1.00$ CG-pp: $SMD_{W}=-0.13$ EG1-CG: $SMD_{b}=-0.12\;(-1.10,\;0.86)$ EG2-CG: $SMD_{b}=1.01\;(-0.03,\;2.06)$
					*Upper-extremity muscle power:* Overhead medicine ball throw test [km/h]	EG1-pp: $SMD_{W}=2.15$ EG2-pp: $SMD_{W}=2.64$ CG-pp: $SMD_{W}=0.43$ EG1-CG: $SMD_{b}=1.32\;(0.24,\;2.40)$ EG2-CG: $SMD_{b}=1.37\;(0.28,\;2.45)$
					*Speed:* 10 m sprint [s]	EG1-pp: $SMD_{W}=1.00$ EG2-pp: $SMD_{W}=-0.057$ CG-pp: $SMD_{W}=-0.017$ EG1-CG: $SMD_{b}=-0.15\;(-1.13,\;0.83)$ EG2-CG: $SMD_{b}=-1.00\;(-2.04,0.04)$
					*Agility:* 505-agility test [s]	EG1-pp: $SMD_{W}=0.80$ EG2-pp: $SMD_{W}=-0.11$ CG-pp: $SMD_{W}=-0.53$ EG1-CG: $SMD_{b}=2.82\;(1.44,\;4.20)$ EG2-CG: $SMD_{b}=2.23\;(0.98,\;3.48)$
Mengyao et al. (43)	30, M; 16–18 years; N/A	EG (*n *= 15): core strength training CG (*n *= 15): regular tennis training	8 wk, 3 d, 24 sessions, 60 min, 180 min 11 exercises, 3 sets of 15 reps	Bird dog and side bridges, back extension, raised upper body and lower body, abdominal crunch and Swiss ball, crunch, plank, lunge, squat, Russian twist on the Swiss ball, bicycle crunch, medicine ball throws	*Agility:* Spider-test [s]	EG-pp: $SMD_{W}=1.27$ CG-pp: $SMD_{W}=0.46$ EG-CG: $SMD_{b}=-0.82\;(-1.56,\;-0.07)$

CG, control group; CMJ, countermovement jump; d, days; EG, experimental group; F, female; ITN, international tennis number; M, male; mo, month; NA/, not available; RM, repetition maximum; SJ, squat jump; *SMD*_b_, between-subject standardized mean differences; *SMDw*, within-subject standardized mean difference; VIFT, velocity of the intermittent fitness test; wk, weeks.

### 3.3. Outcome measures

Fourteen studies (10, 11, 27, 30, 31, 33, 34, 36–38, 40–43) investigated the influence of athletic training on measures of physical fitness and 15 studies (10, 11, 14, 15, 24–26, 28, 29, 32, 33, 35, 37, 39, 40) on parameters of stroke velocity. In terms of physical fitness, nine studies (10, 11, 31, 33, 34, 36, 40–42) examined lower-extremity muscle power and three studies (11, 33, 40) assessed upper-extremity muscle power. Five articles (10, 11, 31, 34, 41) analyzed tennis-specific endurance, eleven articles (11, 27, 30, 33, 34, 36–38, 41–43) investigated agility, nine studies (11, 30, 31, 33, 34, 36, 40–42) evaluated speed, two papers (11, 36) explored balance, three articles (36, 40, 41) studied flexibility, and five studies (10, 11, 14, 40, 41) quantified upper-extremity muscle strength.

### 3.4. Intervention characteristics

In total, 33 different interventions were performed. Athletic training duration ranged from four weeks to nine months with a period of 6–8 weeks being used most frequently (*n *= 18 studies). The players completed two to three sessions of additional athletic training per week. Each training session lasted between 16 and 90 min, although in some studies (*n *= 4) session duration was not specified. On average, nine exercises were performed during each training session, although the respective number ranged from a minimum of three (34) to a maximum of 14 (10) different exercises per session. However, seven studies (15, 24, 25, 28, 29, 38, 39) did not report the number of exercises which were executed during the intervention. In eleven studies (10, 11, 14, 24–26, 28, 32, 35, 36, 42), various strength training programs (e.g., shoulder resistance training, periodized or non-periodized resistance training, single set circuit training, non-linear periodized resistance training) were conducted, six papers (14, 15, 29, 33, 35, 38) investigated the influence of plyometric training (e.g., CMJ, SJ, medicine ball chest pass), three studies (37, 40, 43) analyzed the effectiveness of core training (e.g., plank, dead bug, climbers), and two studies (36, 41) conducted functional training (e.g., burpees, jump squat, agility run, plank, squat, medicine ball throw). In addition, several other interventions were carried out: overweight racket training (15), balance training (e.g., training on unstable underground, unipedal balance exercises) (30), combined explosive strength and repeated sprint training (e.g., plyometric jumps, agility drills, CMJ, 15–20 m sprints) (31), mixed high intensity intermittent runs (34), combined training (e.g., agility, strength, endurance) (39), and agility training (27).

### 3.5. Methodological quality of the included trials

The included studies achieved a PEDro score between 4 and 7 points. Eighteen out of 24 studies achieved the cut-off score of ≥ 6 points, while six studies did not achieve this score. Three studies of these examined young players (36, 39, 43), and three studies explored adult players (11, 32, 38).

### 3.6. Effects of athletic training on measures of physical fitness

#### 3.6.1. Speed

**Figure 2:**
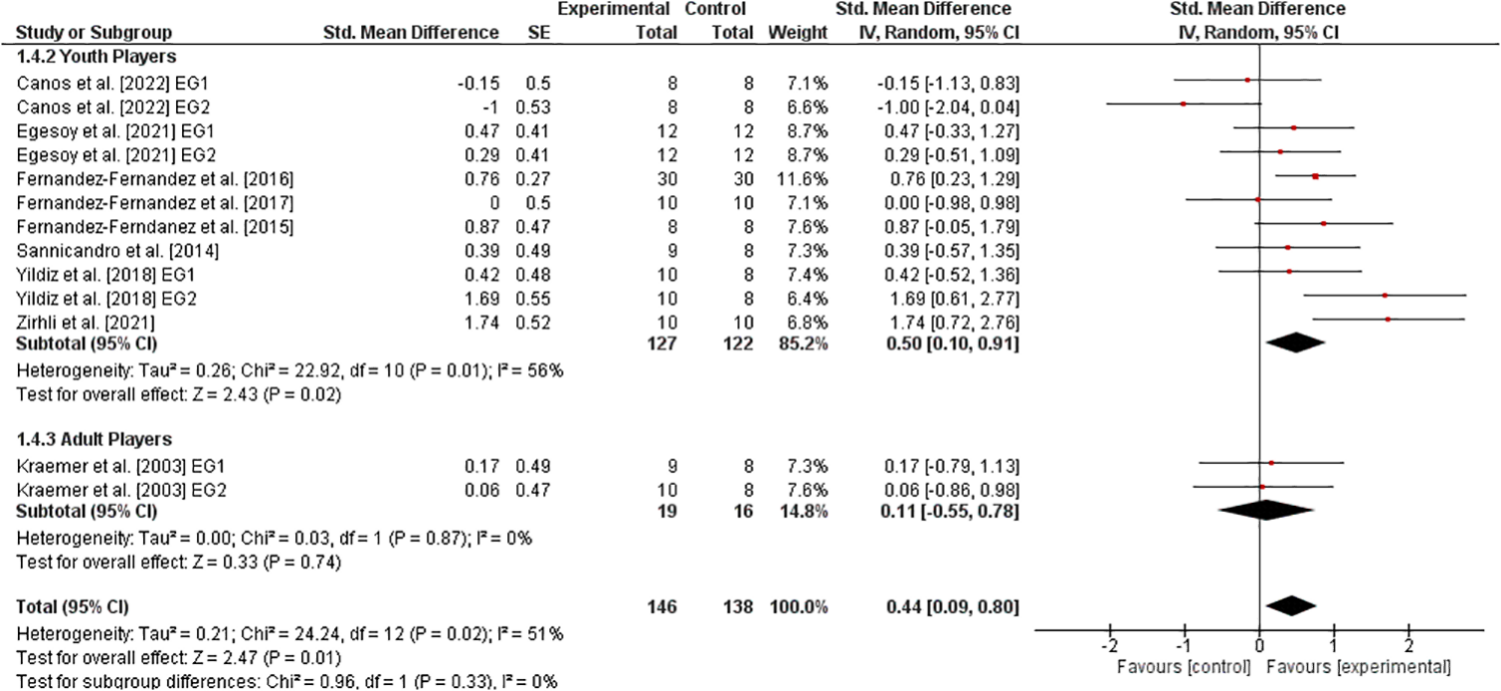
Effects of athletic training on measures of speed (e.g., 20 m sprint time) in healthy youth and adult tennis players. *CI *= confidence interval, *df *= degrees of freedom, *SE *= standard error, *IV *= inverse variance.

Figure 2 shows the effects of athletic training on measures of speed in healthy tennis players. Eight studies (30, 31, 33, 34, 36, 40–42) investigated youth players and one study (11) dealt with adult players. For all players, the weighted mean *SMD*_b_ amounted to 0.44 (9 studies, *I*^2^ = 51%, *Chi*^2^ = 24.2, df=12,p=.02), which is indicative of a small effect favoring the EG. Further, the age-specific sub-analysis revealed a moderate effect in youth $\left(SMD_{b}=0.50\right)$ and a small effect in adult $\left(SMD_{b}=0.11\right)$ players, both in favor of the EG.

#### 3.6.2. Agility

**Figure 3:**
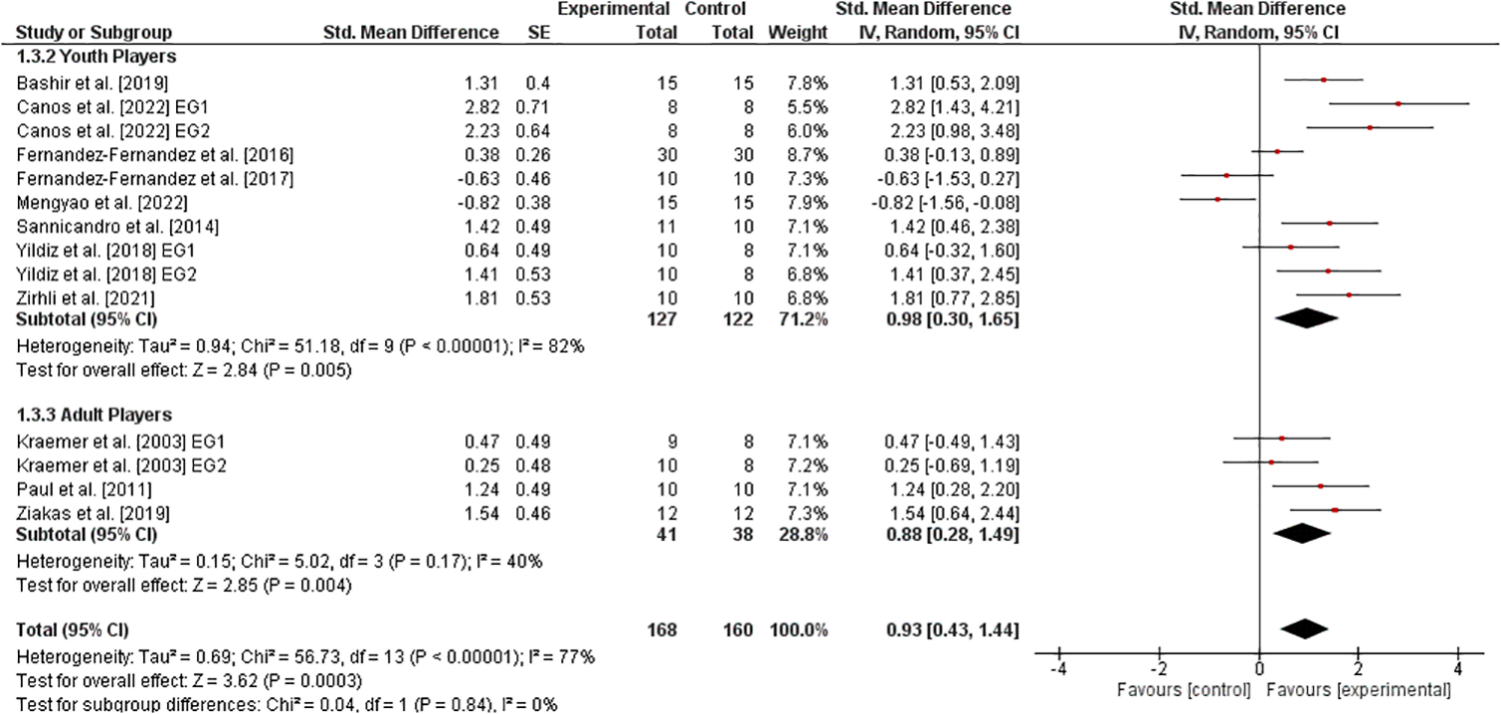
Effects of athletic training on measures of agility (e.g., 5-0-5 test time) in healthy youth and adult tennis players. *CI *= confidence interval, *df *= degrees of freedom, *SE *= standard error, *IV *= inverse variance.

The effects of athletic training on variables of agility in healthy tennis players are displayed in Figure 3. Eight studies (30, 33, 34, 36, 37, 41–43) investigated youth players and three studies (11, 27, 38) analyzed adult players. When considering all players, the weighted mean *SMD*_b_ yielded 0.93 $\left(11\;\mathrm{studies},I^{2}=77\text{\%},\;Chi^{2}=56.73,\;df=13,\;p<0.00001\right)$, indicating a large effect in favor of the EG. In addition, the age-specific sub-analysis showed a large effect in youth $\left(SMD_{b}=0.98\right)$ and in adult $\left(SMD_{b}=0.88\right)$ players, both in favor of the EG.

#### 3.6.3. Lower-extremity muscle power

**Figure 4:**
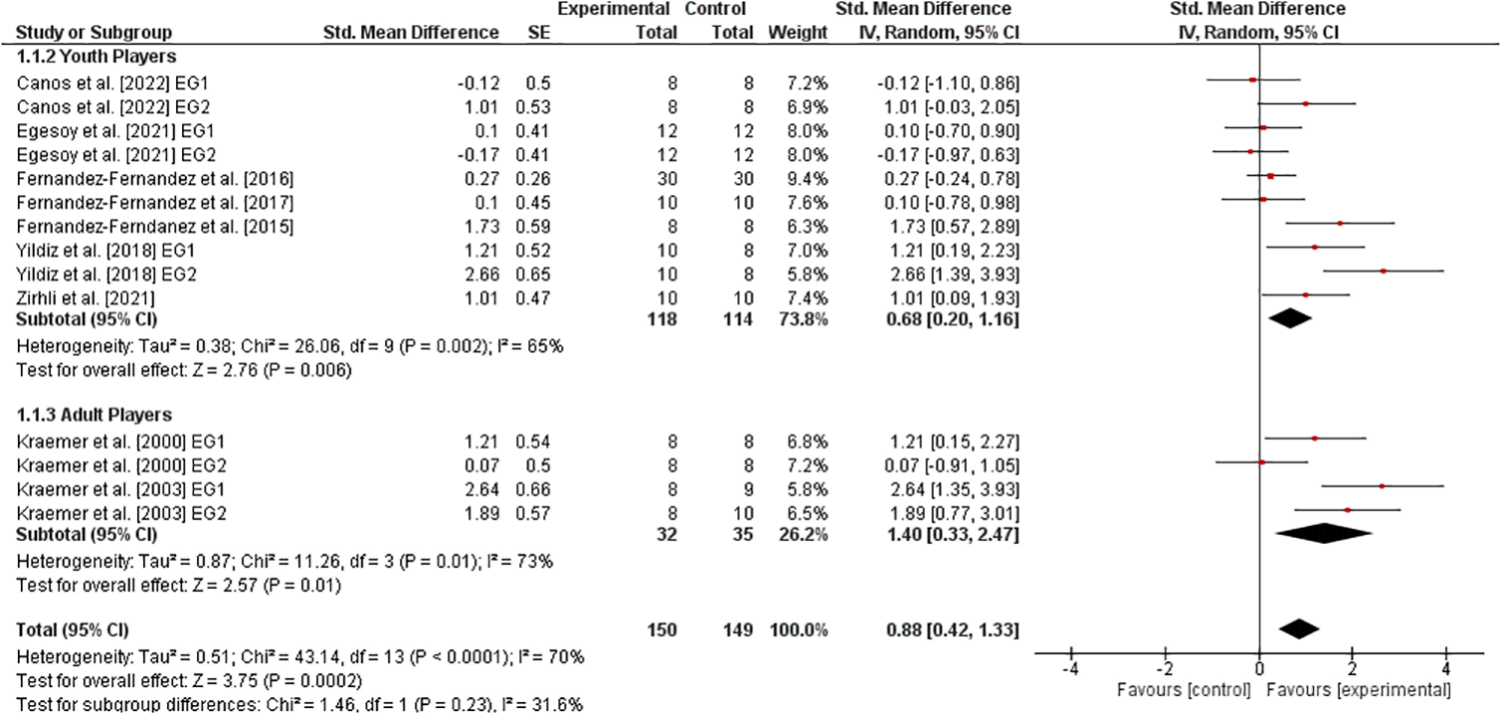
Effects of athletic training on measures of lower-extremity muscle power (e.g., counter movement jump height) in healthy youth and adult tennis players. *CI *= confidence interval, *df *= degrees of freedom, *SE *= standard error, *IV *= inverse variance.

Figure 4 illustrates the effects of athletic training on parameters of lower-extremity muscle power in healthy tennis players. Six studies (31, 33, 34, 36, 40, 41) examined youth players and two studies (10, 11) assessed adult players. In general, the weighted mean *SMD*_b_ was 0.88 (8 studies, $I^{2}=70\text{\%},\;Chi^{2}=43.14,\;df=13,\;p<0.0001)$, which indicates a large effect in favor of the EG. The additionally performed age-specific sub-analysis revealed a moderate effect in youth $\left(SMD_{b}=0.68\right)$ and a large effect in adult $\left(SMD_{b}=1.40\right)$ players, both in favor of the EG.

#### 3.6.4. Upper-extremity muscle power

**Figure 5:**
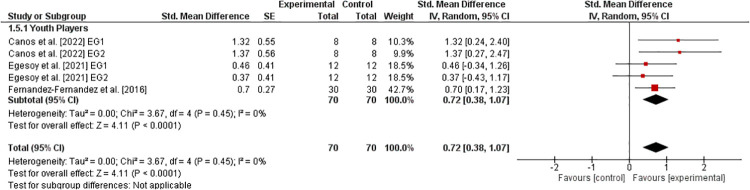
Effects of athletic training on measures of upper-extremity muscle power (e.g., medicine ball throw) in healthy youth tennis players. *CI *= confidence interval, *df *= degrees of freedom, *SE *= standard error, *IV *= inverse variance.

 Figure 5 shows the effects of athletic training on parameters of upper-extremity muscle power in healthy youth tennis players (33, 40, 42). The weighted mean *SMD*_b_ amounted to 0.72 (3 studies, *I*^2^ = 0%, Chi^2^ = 3.67, *df* = 4, p=0.45) indicating a moderate effect in favor of the EG.

#### 3.6.5. Upper-extremity muscle strength

**Figure 6:**
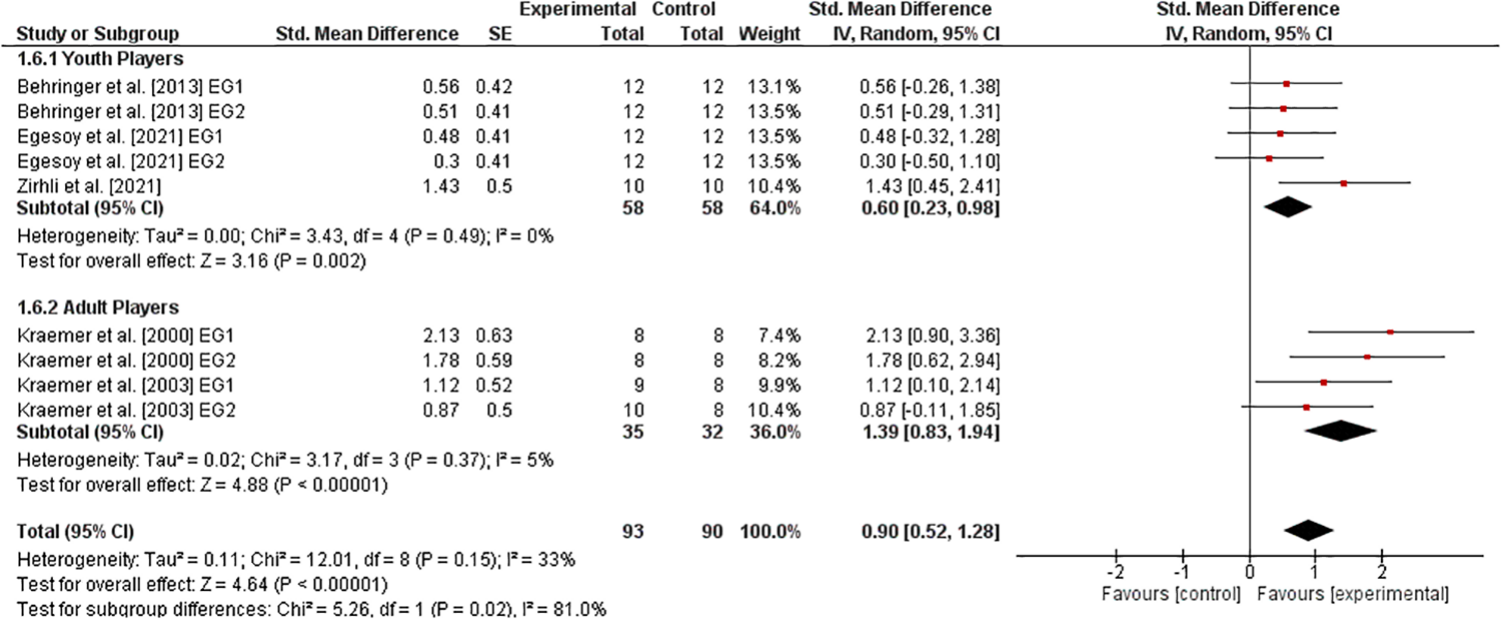
Effects of athletic training on measures of upper-extremity muscle strength (e.g., handgrip strength) in healthy youth and adult tennis players. *CI *= confidence interval, *df *= degrees of freedom, *SE *= standard error, *IV *= inverse variance.

 Figure 6 displays the effect of athletic training on variables of upper-extremity muscle strength in healthy tennis players. Three studies (14, 40, 41) evaluated youth players and two studies (10, 11) analyzed adult players. For all players, the weighted mean *SMD*_b_ of 0.90 (5 studies, *I*^2^ = 33%, *Chi*^2^ = 12.01, df=8,p=0.15) indicates a large effect in favor of the EG. Further, the age-specific sub-analysis revealed a moderate effect in youth $\left(SMD_{b}=0.60\right)$ and a large effect in adult $\left(SMD_{b}=1.39\right)$ players, both in favor of the EG.

#### 3.6.6. Endurance

**Figure 7:**
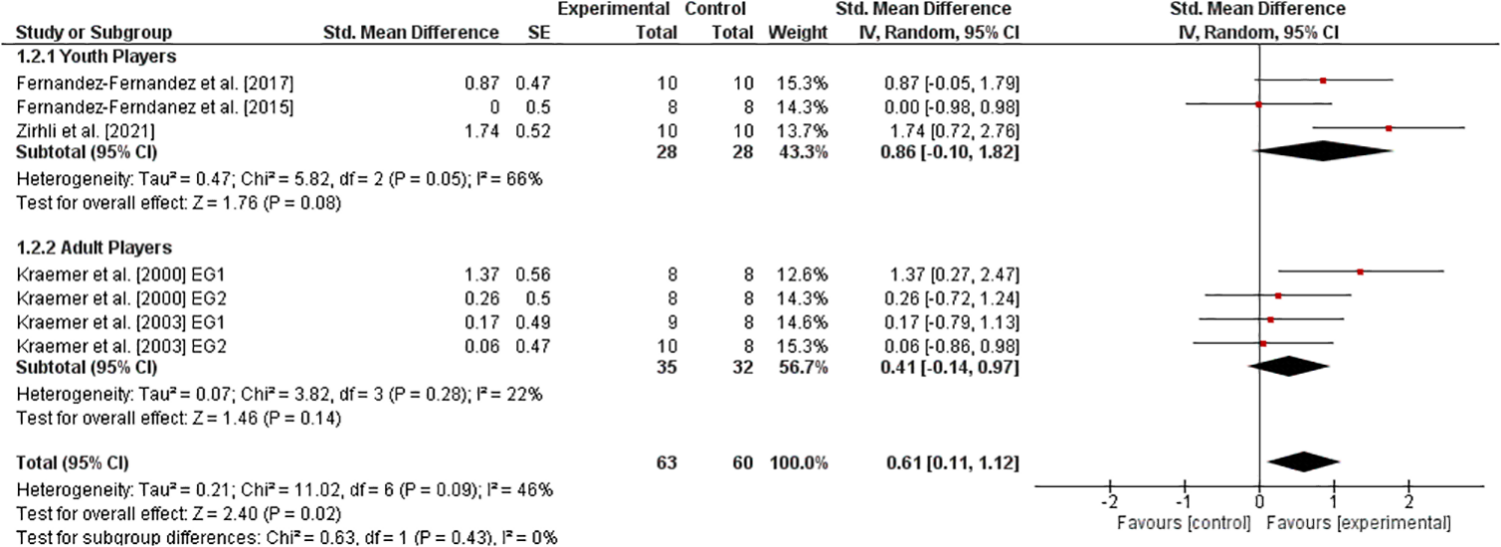
Effects of athletic training on measures of endurance (e.g., VO_2_max) in healthy youth and adult tennis players. *CI *= confidence interval, *df *= degrees of freedom, *SE *= standard error, *IV *= inverse variance.

The effects of athletic training on measures of endurance in tennis athletes are shown in Figure 7. Three studies (31, 34, 41) examined youth players and two studies (10, 11) dealt with adult players. Overall, the weighted mean *SMD*_b_ amounted to 0.61 $\left(4\;\mathrm{studies},\;I^{2}=46\text{\%},\;Chi^{2}=11.02,\;df=6,\;p=0.09\right)$, indicating a moderate effect favoring the EG. Moreover, a large effect was detected in youth $\left(SMD_{b}=0.86\right)$ and a small effect in adult $\left(SMD_{b}=0.41\right)$ players, both in favor of the EG.

#### 3.6.7. Balance

**Figure 8:**
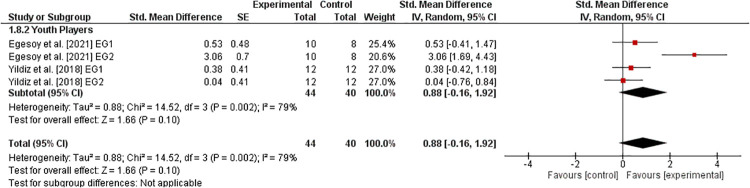
Effects of athletic training on measures of balance (e.g., Y balance test) in healthy youth tennis players. *CI *= confidence interval, *df *= degrees of freedom, *SE *= standard error, *IV *= inverse variance.

Two studies (36, 40) investigated the effects of athletic training on measures of balance performance in healthy youth tennis players (Figure 8). Our analysis revealed a weighted mean *SMD*_b_ of 0.88 (2 studies, *I*^2^ = 79%, *Chi*^2^ = 14.52, df=3,p=0.002) indicating a large effect in favor of the EG.

#### 3.6.8. Flexibility

**Figure 9:**
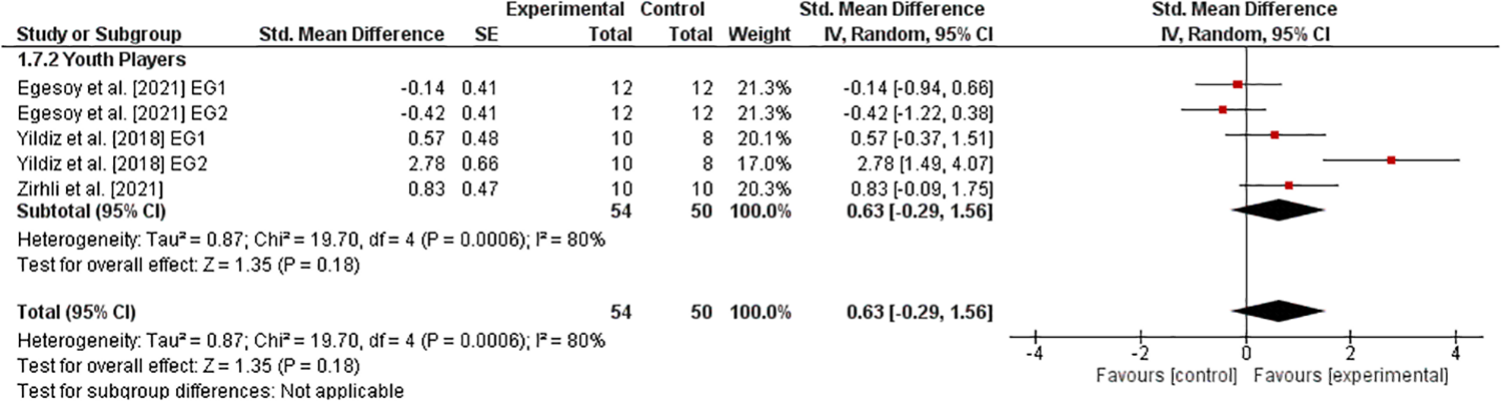
Effects of athletic training on measures of flexibility (e.g., sit-and-reach test) in healthy youth tennis players. *CI *= confidence interval, *df *= degrees of freedom, *SE *= standard error, *IV *= inverse variance.

Three studies (36, 40, 41) examined the effects of athletic training on parameters of flexibility in healthy youth tennis players (Figure 9). The weighted mean *SMD*_b_ amounted to 0.63 (3 studies, *I*^2^ = 80%, *Chi*^2^ = 19.70, *df* = 4, p=0.0006), which indicates a moderate effect in favor of the EG.

### 3.7. Effects of athletic training on measures of stroke velocity

**Figure 10:**
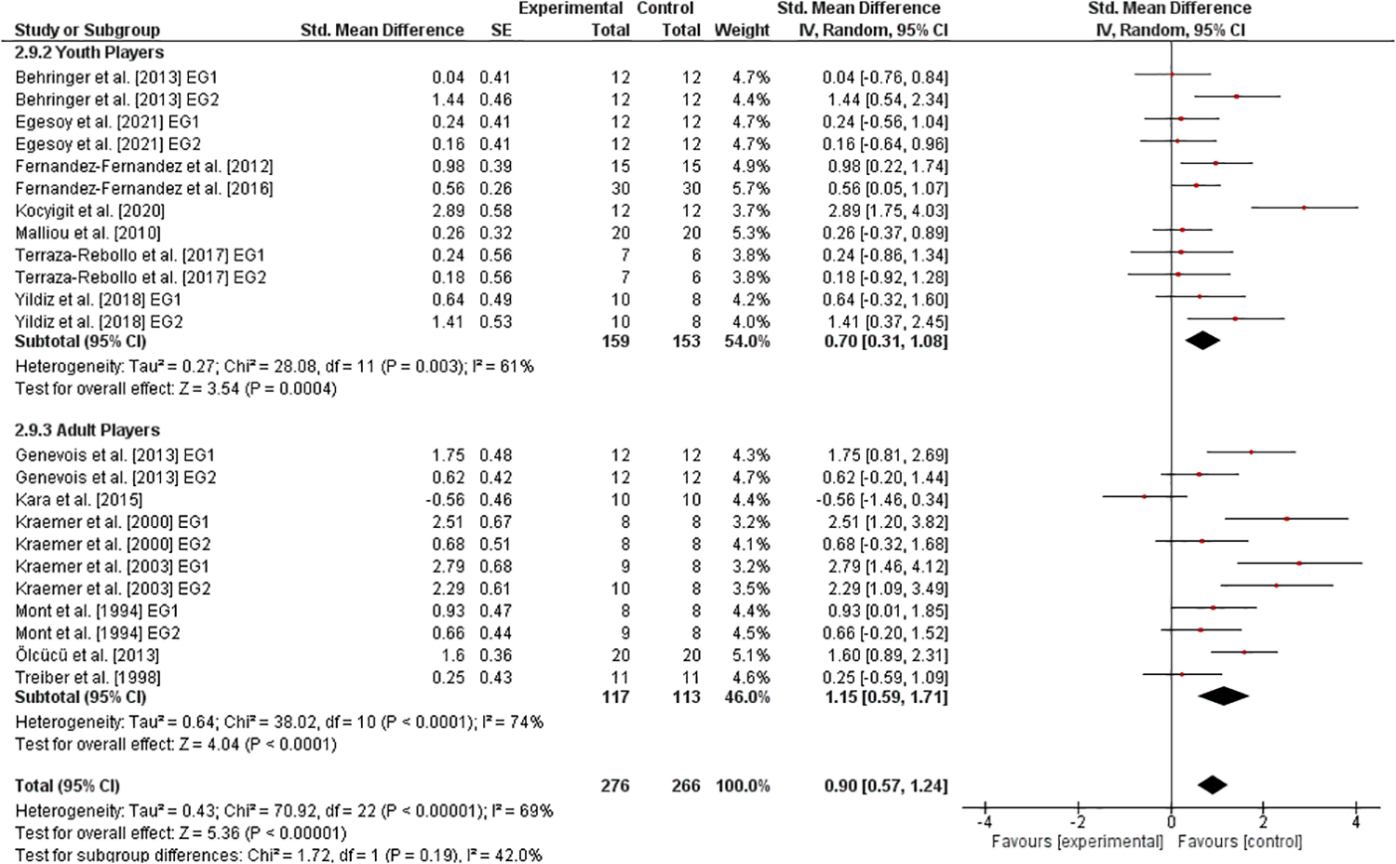
Effects of athletic training on measures of stroke performance (e.g., maximal stroke velocity) in healthy youth and adult tennis players. *CI *= confidence interval, *df *= degrees of freedom, *SE *= standard error, *IV *= inverse variance.

The effects of athletic training on parameters of stroke velocity) in healthy tennis players are shown in Figure 10. Eight studies (14, 26, 28, 33, 35, 36, 39, 40) analyzed youth players and six studies (10, 11, 15, 24, 25, 32) examined adult players. Overall, the analyses yielded a weighted mean *SMD*_b_ of 0.90 $\left(I^{2}=69\text{\%},\;Chi^{2}=70.92\;df=22,\;p<0.00001\right)$ indicating a large effect favoring the EG. Furthermore, large effects in youth $\left(SMD_{b}=0.70\right)$ as well as in adult $\left(SMD_{b}=1.15\right)$ players were detected.

## 4. Discussion

To the best of our knowledge, the present systematic review with meta-analysis is the first to characterize, aggregate, and quantify the effects of athletic training programs on measures of physical fitness and stroke velocity in healthy youth and adult tennis players. Overall, the analysis of the data of 24 studies that met the criteria selection revealed for measures of physical fitness small (speed: *SMD* = 0.44), moderate (endurance: *SMD* = 0.61, upper-extremity muscle power: *SMD* = 0.72, flexibility: *SMD* = 0.63), and large (agility: *SMD* = 0.83, lower-extremity muscle power: *SMD* = 0.88, upper-extremity muscle strength: *SMD* = 0.90, balance: *SMD* = 0.88) effects, all in favor of the EG. For stroke velocity (e.g., maximal and mean stroke velocity), the analyses yielded a large effect of physical training (*SMD* = 0.90) also favoring the EG. Furthermore, the additionally performed sub-analyses showed differences in the effectiveness of athletic training programs on variables of physical fitness and stroke velocity when considering players' age (i.e., youth players: < 18 years vs. adult players: ≥ 18 years).

### 4.1. Effectiveness of athletic training on measures of physical fitness

In line with our hypothesis stating that athletic training will lead to improvements in variables of physical fitness, but the effectiveness will differ with respect to of players' age (i.e., youth vs. adult tennis players), this present systematic review with meta-analysis showed beneficial effects of athletic training on measures of physical fitness in healthy tennis players in favor of the EG, which can be classified as small to large. Specifically, large effects were detected for agility (*SMD* = 0.93), balance (*SMD* = 0.88), lower-extremity muscle power (*SMD* = 0.88), and upper-extremity muscle strength (*SMD* = 0.90), indicating a high trainability (large adaptive reserve) in these physical fitness components.

In terms of agility, the sub-analysis showed a large effect in youth (*SMD* = 0.98) as well as in adult (*SMD* = 0.88) players, suggesting a high adaptive reserve in both age groups. Precisely, agility is mainly composed of two components: (i) change of direction speed (i.e., technique, straight sprinting speed, leg muscle qualities, and anthropometry) and (ii) perceptual and decision-making factors (visual scanning, knowledge of situations, pattern recognition, and anticipation) (44). Due to their complex composition, both components require several years of training to achieve maximum performance (27), which means that training-related adaptations progress can be achieved regardless of age and with the use of different interventions. Precisely, functional training (41), plyometric training (38), and flywheel training (42) were applied and all of them resulted in positive effects on measures of agility. Furthermore, Meckel et al. (45) showed that agility accounted for almost 40% of the players’ ranking (i.e., country's youth tennis players listing). Therefore, from this and the previously reported findings of the present study, it can be deduced that the promotion of agility seems particularly important for success in tennis.

Regarding balance, the large effect (*SMD* = 0.88) refers exclusively to the youth tennis players, since no study was found for adult players. There seems to be a particularly high adaptive reserve due to ongoing growth, maturation, and developmental processes in children and adolescents. Particularly, the neural system is not yet fully matured and thus offers a prominent potential for the promotion of informationally determined physical fitness components like balance (46). In this regard, several original studies (47–49) as well as review articles and meta-analyses (50, 51) have shown significant improvements following balance training on measures of balance and sport-related performance in youth.

With respect to lower-extremity muscle power, the sub-analysis revealed a moderate effect (*SMD* = 0.68) in youth and a large effect (*SMD* = 1.40) in adult players. This indicates that there is a high potential for muscular adaptations at a later stage. In fact, factors favorably influencing the development and training of muscular strength, such as an increase in circulating androgens (e.g., testosterone), are reported for the transition from youth to adulthood (52, 53). In this context, Vrijens (54) showed larger improvements (i.e., isometric strength of the elbow flexors/extensors and knee flexors/extensors) in pubertal (i.e., 16-year-olds) compared to prepubertal participants (i.e., 10-year-olds) following eight weeks (3 times per week) of resistance training. Concerning upper-extremity muscle strength, the sub-analysis yielded similar results, namely a moderate effect (*SMD* = 0.60) in youth and a large effect (*SMD* = 1.39) in adult players. Thus, as for lower-extremity muscle power the same line of argumentation can be applied.

Moderate effects were obtained for upper-extremity muscle power (*SMD* = 0.72), flexibility (*SMD* = 0.63), and endurance (*SMD* = 0.61). Regarding upper-extremity muscle power and flexibility, the moderate effects refer solely to the youth tennis players, as no studies were found for adult players. Thus, both physical fitness components seem to be well trainable in youth tennis players. In this context, using a regression analysis Ulbricht et al. (2) showed that upper-extremity muscle power was the most correlated predictor of tennis performance (i.e., national youth ranking) in female and male elite junior tennis players. Therefore, promoting upper-extremity muscle power seems particularly worthwhile for enhancing tennis performance.

In terms of endurance, the sub-analysis showed a large effect (*SMD* = 0.86) in youth and a small effect (*SMD* = 0.41) in adult players, indicating that the former one seems to have a higher adaptive reserve. Again, it can be argued that processes such as growth, maturation, and development are not yet complete in youth compared to adult players, and the cardiovascular as well as pulmonary system offers a particular potential for the promotion of energetically determined physical fitness components such as endurance (55). In this regard, a recent systematic and meta-analysis (56) revealed beneficial effects of endurance training (i.e., high-intensity interval training) on oxygen consumption, heart rate, repeated sprint ability etc. in young athletes (mean age: 15.5 ± 2.2 years).

A small effect was found for speed (*SMD* = 0.44). However, the sub-analysis showed a small effect (*SMD* = 0.11) only in adult players but a moderate effect (*SMD* = 0.50) in youth players. Most likely, this is because speed is a component that is largely genetically determined (55). Thus, the potential for training-induced adaptations is relatively low. Since the neuronal system, which is responsible for speed-related processes such as the perception, processing, and transmission of information is not yet fully mature in children and adolescents compared to adults, youth players seem to have more possibilities for training-related adaptation, which may explain their moderate effect (57).

### 4.2. Effectiveness of athletic training on measures of stroke velocity

In accordance with our hypothesis stating that athletic training will result in enhancements in stroke velocity, but the effectiveness will differ depending on players' age (i.e., youth vs. adult tennis players), we identified large effects (*SMD* = 0.90) of athletic training on stroke velocity in healthy tennis players in favor of the EG. However, the sub-analysis showed a large effect (*SMD* = 1.15) only in adult players but a moderate effect (*SMD* = 0.70) in youth players. Thus, both age groups seem to have a good adaptive potential for the promotion of stroke velocity, which is even higher in adult players. In terms of adult players, the interventions used ranged from plyometric training (29) over medicine ball training (14) to periodized strength training (10, 11) (e.g., crunches, back extensions, split squats), with non-linear periodized resistance being particularly effective. In accordance to this, the German Tennis Confederation (58) recommends to improve stroke velocity by using athletic exercises such as multi-directional jumps, medicine ball throws, and core strengthening. For youth players, the German Tennis Confederation (58) recommends improving stroke velocity especially by practicing stroke techniques, as evidence exists that technical demands and the underlying motor skills and cognitive processes are acquired through several years of practice (59). In addition, athletic exercises should be performed. In the present systematic review and meta-analysis the intervention used ranged from plyometric training (14) over functional training (36) (e.g., squat, plank, dead bug) to combined training (39) (including strength, speed, agility, and endurance exercises) with combined training being particularly effective.

### 4.3. Limitations

This systematic review with meta-analysis has a few limitations. The used methodology varied between the included studies in terms of players' characteristics (age, sex, performance level), assessments (tests, outcomes), and interventions (modalities like duration, frequency, volume of training etc.) which is reflected in a trivial to considerable heterogeneity between studies. Thus, future studies should apply instrumented assessment methods (i.e., biomechanical tests using force plates, plantar pressure devices etc.) in addition to the frequently used field-based tests to reduce the variability in effect estimates. Further, the included studies represent healthy tennis players in the age range of 6–42 years, thus no statement can be made especially about master athletes. Moreover, of the 24 included studies, three and eleven studies examined only women and men, respectively. Therefore, no sex-specific analyses could be performed.

## 5. Conclusions

The systematic review and meta-analysis characterized, aggregated, and quantified the effects of athletic training programs on measures of physical fitness and stroke velocity in healthy tennis players. For measures of physical fitness, we detected small (speed), moderate (endurance, upper-extremity muscle power, flexibility), and large (agility, lower-extremity muscle power, upper-extremity muscle strength, balance) effects, all in favor of the EG. In addition, a large effect also favoring the EG was found for parameters of stroke velocity. This indicates that athletic training is effective to varying degrees and this is further influenced by players' age (i.e., youth players: < 18 years vs. adult players: ≥ 18 years). For both age groups, we therefore conclude that further research is needed to investigate optimal training regimes in order to enlarge the effectiveness especially for those fitness components that showed small- to moderate-sized changes.

## 6. Practical applications

The results of the present systematic review with meta-analysis reveal implications for practitioners. In terms of physical fitness outcomes, large effects and thus a high potential for training-induced adaptations were found in youth players with respect to agility, balance and endurance, and in adult players with respect to agility, lower-extremity muscle power and upper-extremity muscle strength. This age specificity in trainability should therefore be considered when designing programs for long-term athlete development. In terms of stroke velocity, large effects were detected in adult and moderate effects in youth players. This suggests similar trainability in both age categories, according to which programs to train stroke techniques should start in adolescence and continue throughout adulthood.

## Data Availability

The original contributions presented in the study are included in the article/Supplementary Material, further inquiries can be directed to the corresponding author/s.

## References

[1] Kramer T Huijgen BC Elferink-Gemser MT Visscher C. A longitudinal study of physical fitness in elite junior tennis players. Pediatr Exerc Sci. (2016) 28:553–64. 10.1123/Pes.2016-0022 27705537

[2] Ulbricht A Fernandez-Fernandez J Mendez-Villanueva A Ferrauti A. Impact of fitness characteristics on tennis performance in elite junior tennis players. J Strength Cond Res. (2016) 30:989–98. 10.1519/Jsc.0000000000001267 26605803

[3] Brechbuhl C Girard O Millet GP Schmitt L. Differences within elite female tennis players during an incremental field test. Med Sci Sports Exerc. (2018) 50:2465–73. 10.1249/Mss.0000000000001714 29975301

[4] Elliott BC Ackland TR Blanksby BA Bloomfield J. A prospective study of physiological and kinanthrompoemtric indicators of junior tennis performance. Aust J Sport Sci. (1990) 22:87–92.

[5] Girard O Micallef J-P Millet GP. Lower-Limb activity during the power serve in tennis: effects of performance level. Med Sci Sports Exerc. (2005) 37:1021. 10.1249/01.Mss.0000171619.99391.Bb 15947729

[6] Sánchez-Pay A Ramón-Llin J Martínez-Gallego R Sanz-Rivas D Sánchez-Alcaraz BJ Frutos S. Fitness testing in tennis: influence of anthropometric characteristics, physical performance, and functional test on serve velocity in professional players. Plos One. (2021) 16:E0259497. 10.1371/Journal.Pone.0259497 34843515 PMC8629317

[7] Kurtz JA Grazer J Alban B Marino M. Ability for tennis specific variables and agility for determining the universal tennis ranking (utr). Sports J. (2019) 4(2):1–16.

[8] Kolman NS Huijgen BC Visscher C Elferink-Gemser MT. The value of technical characteristics for future performance in youth tennis players: a prospective study. Plos One. (2021) 16:E0245435. 10.1371/Journal.Pone.0245435 33439916 PMC7806163

[9] Lambrich J Muehlbauer T. Physical fitness and stroke performance in healthy tennis players with different competition levels: a systematic review and meta-analysis. Plos One. (2022) 17:1–15. 10.1371/Journal.Pone.0269516 PMC9165775 35657986

[10] Kraemer WJ Ratamess N Fry AC Triplett-Mcbride T Koziris LP Bauer JA Influence of resistance training volume and periodization on physiological and performance adaptations in collegiate women tennis players. Am J Sports Med. (2000) 28:626–33. 10.1177/03635465000280050201 11032216

[11] Kraemer WJ Hakkinen K Triplett-Mcbride NT Fry AC Koziris LP Ratamess NA Physiological changes with periodized resistance training in women tennis players. Med Sci Sports Exerc. (2003) 35:157–68. 10.1097/00005768-200301000-00024 12544650

[12] Xiao W Geok SK Bai X Bu T Norjali Wazir MR Talib O Effect of exercise training on physical fitness among young tennis players: a systematic review. Front Public Health. (2022) 10:843021. 10.3389/Fpubh.2022.843021 35309192 PMC8924058

[13] Malina RM Bouchard C Bar-Or O. Growth, maturation, and physical activity. Champaign, il: Human Kinetics (2004). 712.

[14] Behringer M Neuerburg S Matthews M Mester J. Effects of two different resistance-training programs on mean tennis-serve velocity in adolescents. Pediatr Exerc Sci. (2013) 25:370–84. 10.1123/Pes.25.3.370 23986524

[15] Genevois C Frican B Creveaux T Hautier C Rogowski I. Effects of two training protocols on the forehand drive performance in tennis. J Strength Cond Res. (2013) 27:677–82. 10.1519/Jsc.0b013e31825c3290 22592176

[16] Colomar J Corbi F Baiget E. Improving tennis serve velocity: review of training methods and recommendations. Strength Cond J. (2022):1–10. 10.1519/Ssc.0000000000000733. [Epub ahead of print].

[17] Moher D Liberati A Tetzlaff J Altman DG. Preferred reporting items for systematic reviews and meta-analyses: the prisma statement. Ann Intern Med. (2009) 151:264–9. W64. 10.7326/0003-4819-151-4-200908180-00135 19622511

[18] Farrell C. Turgeon. Normal Versus Chronic Adaptations To Aerobic Exercise (2021). Available From: Https://Www.Ncbi.Nlm.Nih.Gov/Books/Nbk572066/#_Nbk572066_Pubdet_ 34283432

[19] Maher CG Sherrington C Herbert RD Moseley AM Elkins M. Reliability of the pedro scale for rating quality of randomized controlled trials. Phys Ther. (2003) 83:713–21. 10.1093/ptj/83.8.713 12882612

[20] Deeks JJ Higgins JP. Statistical algorithms in review manager 5, In: *Statistical Methods Group of The Cochrane Collaboration* (2010). pp. 1–11.

[21] Cohen J. Statistical power analysis. Curr Dir Psychol Sci. (1992) 1:98–101. 10.1111/1467-8721.Ep10768783

[22] Deeks Jonathan J Higgins JP Altman DG. “Analysing data and undertaking meta-analyses,”. In: JPT Higgins S Green, Editors. Cochrane handbook for systematic reviews of interventions. Chichester: The Cochrane Collaboration (2008).

[23] Higgins JP Thompson SG. Quantifying heterogeneity in A meta-analysis. Stat Med. (2002) 21:1539–58. 10.1002/Sim.1186 12111919

[24] Mont MA Cohen DB Campbell KR Gravare K Mathur SK. Isokinetic concentric versus eccentric training of shoulder rotators with functional evaluation of performance enhancement in elite tennis players. Am J Sports Med. (1994) 22:513–7. 10.1177/036354659402200413 7943517

[25] Treiber FA Lott J Duncan J Slavens G Davis H. Effects of theraband and lightweight dumbbell training on shoulder rotation torque and serve performance in college tennis players. Am J Sports Med. (1998) 26:510–5. 10.1177/03635465980260040601 9689369

[26] Malliou P Papadimitriou D Malliou V Beneka A Pafis G Katsikas C The effect of strength training on tennis service performance of junior tennis players. Exerc Qual Life. (2011) 3(1):31–40.

[27] Paul M Biswas S Gaurang S Sandhu J. Effect of agility training on tennis. J Medi Sci Tennis. (2011) 6:21–5.

[28] Fernandez-Fernandez J Zimek R Wiewelhove T Ferrauti A. High-Intensity interval training vs. Repeated-sprint training in tennis. J Strength Cond Res. (2012) 26:53–62. 10.1519/Jsc.0b013e318220b4ff 21904233

[29] Ölcücü B Erdil G Altinkök M. Evaluation of the effect of plyometric exercises on the speed of the ball and the hitting percentage during A service. Beden Eğitimi Ve Spor Bilimleri Dergisi. (2013) 7:48–59.

[30] Sannicandro I Cofano G Rosa RA Piccinno A. Balance training exercises decrease lower-limb strength asymmetry in young tennis players. J Sports Sci Med. (2014) 13:397–402. PMID: 24790496, PMCID: PMC3990896 24790496 PMC3990896

[31] Fernandez-Fernandez J Sanz-Rivas D Kovacs MS Moya M. In-Season effect of A combined repeated sprint and explosive strength training program on elite junior tennis players. J Strength Cond Res. (2015) 29:351–7. 10.1519/Jsc.0000000000000759 25436636

[32] Kara E Aksit T Ozkol M. Effects of 6 week tennis specific exercises program on service velocity. Tjse. (2015) 17:71. 10.15314/Tjse.2015112541

[33] Fernandez-Fernandez J Saez De Villarreal E Sanz-Rivas D Moya M. The effects of 8-week plyometric training on physical performance in young tennis players. Pediatr Exerc Sci. (2016) 28:77–86. 10.1123/Pes.2015-0019 26252503

[34] Fernandez-Fernandez J Sanz D Sarabia JM Moya M. The effects of sport-specific drills training or high-intensity interval training in young tennis players. Int J Sports Physiol Perform. (2017) 12:90–8. 10.1123/Ijspp.2015-0684 27140481

[35] Terraza-Rebollo M Baiget E Corbi F Planas Anzano A. Effects of strength training on hitting speed in young tennis players. Rimcafd. (2017) 66:349–366. 10.15366/Rimcafd2017.66.009

[36] Yildiz S Pinar S Gelen E. Effects of 8-week functional vs. Traditional training on athletic performance and functional movement on prepubertal tennis players. J Strength Cond Res. (2019) 33:651–61. 10.1519/Jsc.0000000000002956 30431536

[37] Bashir SF Nuhmani S Dhall R Muaidi QI. Effect of core training on dynamic balance and agility among Indian junior tennis players. J Back Musculoskelet Rehabil. (2019) 32:245–52. 10.3233/Bmr-170853 30248028

[38] Ziagkas E Zilidou VI Loukovitis A Politopoulos N Douka S Tsiatsos T. “The effects of 8-week plyometric training on tennis agility performance, improving evaluation throw the makey makey,”. In: Me Auer Tsiatsos T, Editors. The challenges of the digital transformation in education: Proceedings of the 21st international conference on interactive collaborative learning (Icl2018) - volume 2. Cham: Springer International Publishing (2019). P. 280–6.

[39] Kocyigit B Akin S Sentürk A. The effects of combined trainings on tennis serve speed in tennis players. Turkiye Klinikleri J Sports Sci. (2020) 12:137–46. 10.5336/Sportsci.2019-70168

[40] Egesoy H Oksuzoglu AY Ilhan A. The effects of static and dynamic core training on some motoric characteristics and tennis service velocity on tennis athletes. Ijlpr. (2021) 11(15):296–305.

[41] Zirhli O Demirci N. The influence of functional training on biomotor skills in girl tennis players aged 10–12. Bjhpa. (2020) 12:33–45. 10.29359/Bjhpa.12.4.04

[42] Canós J Corbi F Colomar J Cirer-Sastre R Baiget E. Effects of isoinertial or machine-based strength training on performance in tennis players. Biol Sport. (2022) 39:505–13. 10.5114/Biolsport.2022.107020 35959344 PMC9331344

[43] Mengyao C Seung-Soo B. Effects of core strength training on specialized sports abilities and core stability of adolescent tennis players. Front Sport Res. (2022) 4:14–19. 10.25236/Fsr.2022.040403

[44] Sheppard JM Young WB. Agility literature review: classifications, training and testing. J Sports Sci. (2006) 24:919–32. 10.1080/02640410500457109 16882626

[45] Meckel Y Hophy A Dunsky A Eliakim A. Relationships between physical characteristics and ranking of young tennis players. Cejssm. (2015) 2:5–12.

[46] Shumway-Cook A Woollacott MH. The growth of stability: postural control from A development perspective. J Mot Behav. (1985) 17:131–47. 10.1080/00222895.1985.10735341 15140688

[47] Schedler S Tenelsen F Wich L Muehlbauer T. Effects of balance training on balance performance in youth: role of training difficulty. Bmc Sports Sci Med Rehabil. (2020) 12:71. 10.1186/S13102-020-00218-4 33292455 PMC7684745

[48] Pau M Loi A Pezzotta MC. Does sensorimotor training improve the static balance of young volleyball players? Sports Biomech. (2012) 11:97–107. 10.1080/14763141.2011.637126 22518948

[49] Heleno LR Da Silva RA Shigaki L Araújo CG Coelho Candido CR Okazaki VH Five-Week sensory motor training program improves functional performance and postural control in young male soccer players - A blind randomized clinical trial. Phys Ther Sport. (2016) 22:74–80. 10.1016/J.Ptsp.2016.05.004 27620862

[50] Gebel A Lesinski M Behm DG Granacher U. Effects and dose-response relationship of balance training on balance performance in youth: a systematic review and meta-analysis. Sports Med. (2018) 48:2067–89. 10.1007/S40279-018-0926-0 29736728

[51] Gebel A Prieske O Behm DG Granacher U. Effects of balance training on physical fitness in youth and young athletes: a narrative review. Strength Cond J. (2020) 42:35–44. 10.1519/Ssc.0000000000000548

[52] Round JM Jones DA Honour JW Nevill AM. Hormonal factors in the development of differences in strength between boys and girls during adolescence: a longitudinal study. Ann Hum Biol. (1999) 26:49–62. 10.1080/030144699282976 9974083

[53] Blimkie CJR. Age and sex associated variation in strength during childhood: anthropometric, morphologic, neurologic, biomechanical, endcrinologic, and physical activity correlates. In: CV Gisolfi DR Lamb, Editors. Perspectives in exercise science and sports medicine. Indianapolis: Benchmark (1989). pp. 99–163.

[54] Vrijens J. “Muscle strength development in the Pre- and post-pubescent age,”. In: Borms J, Editor. Pediatric work physiology: with 47 tables. Basel: Karger (1978). P. 152–8.

[55] Hoffman J. Physiological aspects of sport training and performance. Champaign, il: Human Kinetics (2014). 505.

[56] Engel FA Ackermann A Chtourou H Sperlich B. High-Intensity interval training performed by young athletes: a systematic review and meta-analysis. Front. Physiol. (2018) 9:1012. 10.3389/Fphys.2018.01012 30100881 PMC6072873

[57] Bawa P. Neural development in children: a neurophysiological study. Electroencephalogr Clin Neurophysiol. (1981) 52:249–56. 10.1016/0013-4694(81)90054-7 6169502

[58] Eberhard K Fratzke G Jansen E Januschke J Krelle K Spreckels C. *Rahmentrainingskonzeption Des Deutscher Tennis Bund E.V.: Training Methodological Framework Of The German Tennis Federation* (2019).

[59] Yarrow K Brown P Krakauer JW. Inside the brain of an elite athlete: the neural processes that support high achievement in sports. Nat Rev Neurosci. (2009) 10:585–96. 10.1038/Nrn2672 19571792

